# Human skeletal stem cell aging

**DOI:** 10.18632/aging.104034

**Published:** 2020-09-14

**Authors:** Thomas H. Ambrosi, L. Henry Goodnough, Charles K.F. Chan

**Affiliations:** 1 Institute for Stem Cell Biology and Regenerative Medicine, Stanford University School of Medicine, Stanford, CA 94305, USA; 2 Department of Surgery, Stanford University School of Medicine, Stanford, CA 94305, USA; 3 Department of Orthopaedic Surgery, Stanford Hospital and Clinics, Stanford, CA 94305, USA

**Keywords:** human skeletal stem cells, geriatric fractures, aging, regeneration, sexual dimorphism

The world’s population is aging at an alarming rate, and the incidence of osteoporosis is skyrocketing. One major consequence of the decline in skeletal health is increased fracture risk in patients 65 years and older. Osteoporosis-related fractures are predicted to result in more than $25 billion in annual health care costs by 2025. For elderly patients, who demonstrate poor capacity for regeneration and a limited physiologic reserve, surgery has increased complication rates and higher risk of failed healing outcome which is potentially devastating and can result in morbidity and mortality [1]. Current medical therapies are either systemic anabolic or anti-resorptive medications with limited efficacy and major side effects. Aging related bone loss and decline in regenerative potential underlies a detrimental shift of balanced bone formation and resorption dynamics. However, there is very little known on how age-related changes affect the specific skeletal stem cells (SSC) that form mature osteogenic cells in bone tissues.

Stem cell therapy has emerged as a potent new strategy to repair and replace injured tissue. Readily available bone marrow aspirates and adipose tissue have been used as a source for the isolation, study, and transplantation of bone forming cell populations. Despite often being called ‘stem cells’ the isolation of these cells has been largely retrospective in nature and relying on inexact separation criteria such as plastic adherence which produces highly variable and heterogeneous cellular mixtures of multiple tissue types including bone, blood vessels, blood, fat, and fibroblast cells which makes treatment outcomes difficult to evaluate or predict [2]. In contrast, we have recently sought to develop highly specific methods for purifying distinct stem cell types based on differential surface antigen expression using fluorescent conjugated antibodies through FACS (Fluorescence Activated Cell Separation) [3]. Using FACS, our group recently identified a skeletal stem cell (SSC) in both mice and humans. These SSCs are the self-renewing multi-potent stem cells that clonally generate all the lineage-restricted progenitors of the skeleton including bone, cartilage, and the stromal compartment that supports hematopoiesis. This approach enables bona fide SSCs to be specifically purified for functional testing and molecular analysis.

In our recent study, we applied this methodology to isolate human fracture derived SSCs (hSSC) from a broad cohort of patients to test if changes in SSC function were associated with patient age [4]. We prospectively isolated hSSCs from 61 patients (13-94 years) who underwent open surgery for fracture fixation. Interestingly, we found that independent of age and skeletal site, hSSCs accumulated at the fracture site over time. Next, we expanded freshly isolated hSSCs and conducted colony-forming unit assays as well as well-established differentiation assays once the SSC-derived cells reached sub-confluency. These assays revealed that hSSCs from older patients possessed an impaired osteochondrogenic capacity while maintaining similar clonogenicity. Notably, hSSCs from females displayed an exacerbated age-related defect, and postmenopausal females are one of the major subgroups at risk for fragility fractures. Since sexual dimorphic hormonal differences exist, hSSC aging could also be predominantly driven by 5extrinsic rather than intrinsic mechanisms [5]. For instance, exposure to pro-inflammatory stimuli might also alter SSC function by altering epigenetic regulators thereby turning a switch from a young to a geriatric state [6]. Similarly, evidence supports a progressive chronological aging process in stem cells. Future studies utilizing single cell approaches, such as barcoded single cell RNA-sequencing, could explore these hypotheses, as well as the possibility of “skewed clonal skeletogenesis” resulting from somatic mutations, similar to what has been recently been reported in ‘CHIP’ (Clonal Hematopoiesis of Indeterminate Potential) in the hematopoietic compartment [7]. Together with our observation that hSSC prevalence does not seem to be altered with age the latter theory might be supportive of a scenario in humans whereby the lack of osteochondrogenic capacity with age is rather related to lineage skewing towards stromal/fibroblastic fates.

We analyzed genetic regulation of aged SSC traits such as fibrogenicity by performing gene expression analysis on purified hSSCs. We utilized microarray analysis to compare global gene expression in purified hSSCs from young versus aged patients along with corresponding functional evaluation in vitro. Consistent with age-related functional changes, we found downregulation of genes related to bone formation in aged hSSCs. Moreover, aged hSSCs upregulated genes that are related to fibroblast-like extracellular matrix secretion and cellular senescence. Curiously, we also observed that the histone deacetylase Sirtuin1 was significantly downregulated in geriatric hSSCs, which hints at epigenetic mechanisms underlying hSSC aging. We showed that specific blockade of Sirt1 in young hSSCs impairs osteogenic potential in vitro. Excitingly, Resveratrol and a Sirtuin1-specific small molecule restored mineralization capacity in impaired hSSCs thus providing a rationale for future translational strategies.

**Figure 1:**
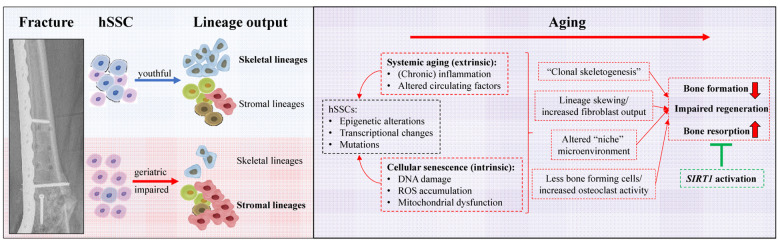
**Summary of human skeleteal stem cell based age-related alterations leading to bone loss and impaired regenerative capacity.** A young functional hSSC is clonally diverse and abundantly gives rise to cell of the skeletal lineages as well as bone marrow supporting stroma. During aging lineage output is skewed which could underlie cellular senescence due to chronological aging or changes forced by an altered microenvironment. Future studies have to explore if aging of hSSCs, similar, to hematopoiesis, leads to clonal expansion of clones with specific characteristics, e.g. preferred fibroblast lineage generation. As a consequence of shifted differentiation to specific stromal lineages not only bone formation is impaired but also bone resorption is increased. Some of these changes might be the consequence of the loss of *SIRT1* expression and reactivation could be a potent therapeutic means to restore youthful hSSC function and thereby reverse age-related bone loss.

In summary, our report of an association between patient age and hSSC function opens new possibilities to identify diagnostic, prognostic, and therapeutic strategies for poor fracture healing and potentially even the preservation of youthful bone health and prevention of skeletal injuries. Targeted molecular therapies that inhibit cellular senescence pathways may reverse age-related fracture phenotypes without broad off-target systemic side effects of current regimens. Continued investigation of larger groups of patients will reveal if simple in vitro assays of hSSCs, or antigens associated with aged hSSCs might serve as a prognostic tool to predict healing outcome of fractures. Furthermore, analyzing hSSCs isolated from nonunions will yield insights into the involvement of hSSCs in the etiology of failed fracture healing. A more comprehensive approach including the analysis of all cells present at the fracture site might also help to elucidate the role of the stem cell microenvironment or “niche” in facilitating proper bone regeneration (Figure 1).
